# Scoping Review on Traditional Fermented Cereals as Catalysts of Precision Nutrition: Mapping Evidence on Probiotic Activity, Gut Microbiota Modulation, and Human Health in Sub-Saharan Africa

**DOI:** 10.3390/biology15181582

**Published:** 2026-09-09

**Authors:** Gbeminiyi Olamiti

**Affiliations:** Department of Food Science and Technology, Faculty of Science, Engineering and Agriculture, University of Venda, Thohoyandou 0950, South Africa; gbeminiyi.olamiti@univen.ac.za

**Keywords:** traditional fermented cereals, lactic acid bacteria, yeasts, probiotics, gut microbiota, precision nutrition, Sub-Saharan Africa

## Abstract

Traditional fermented cereals are affordable, embedded foods that may provide viable microorganisms, fermentable substrates, and bioactive metabolites that can influence the gut microbiota and human physiology. However, evidence linking indigenous cereal fermentations in Sub-Saharan Africa (SSA) to microbiome-mediated health outcomes and precision nutrition applications remains limited. This scoping review mapped the types of traditional fermented cereal foods studied in SSA; their microbial communities and probiotic attributes; the reported effects on gut microbiota composition and function; the documented health outcomes; and the geographic gaps. The review followed the Arksey and O’Malley framework, subsequent methodological refinements, and the PRISMA-ScR reporting guideline. Searches were conducted in Scopus, PubMed, Web of Science, ScienceDirect, and Google Scholar from 2000 to mid-2026, complemented by citation searching. Eligible human, animals, and in vitro studies examined SSA-fermented cereal products and reported microbial, probiotic, gut microbiota, metabolite, or health-related outcomes. After deduplication and screening, 53 studies were included. The evidence was concentrated in SSA, particularly in cereal-based foods. Lactic acid bacteria and yeasts dominated reported fermentation communities, but strain-level validation, dose definition, and controlled human studies were uncommon. Evidence for direct gut–microbiota modulation and clinically important health effects was substantially weaker than evidence for food-level microbial diversity, in vitro probiotic properties, and improved nutrient bioaccessibility. Overall, traditional fermented cereals in sub-Saharan Africa demonstrate promising microbial, nutritional, and functional properties, but current evidence remains heterogeneous and insufficient to establish consistent effects on gut microbiota and human health. Further well-designed human studies integrating dietary, microbiome, and metabolic outcomes are needed to clarify their potential role in precision nutrition.

## Simple Summary

Traditional fermented cereal foods are widely consumed across sub-Saharan Africa and may contribute to better nutrition and health. However, evidence of their effects on human health remains scattered and varies in strength. This scoping review examined what is currently known about fermented cereals, the beneficial microorganisms they contain, their interaction with the microorganisms living in the gut, and their potential role in personalised nutrition. The findings show that fermentation can improve the nutritional value, digestibility, safety, and functional properties of cereal foods, and may also influence the composition of gut microorganisms. Sorghum and millet were the most widely studied cereals, whereas evidence of other, less commonly studied cereals was limited. Most of the available evidence came from laboratory and animal studies, with relatively few human studies, many of which had design limitations. Therefore, although fermented cereals show promising potential for supporting healthy diets and gut wellbeing, stronger human research is needed before specific health benefits can be confirmed. Overall, the findings highlight traditional fermentation as a valuable starting point for developing affordable, culturally appropriate, and locally available foods that may contribute to improved nutrition and health across sub-Saharan Africa.

## 1. Introduction

Traditional fermented cereals have supported food security, cultural identity, and rural livelihoods across Sub-Saharan Africa (SSA) for centuries. Spontaneous fermentation transforms cereals such as maize, sorghum, pearl millet, finger millet, teff, fonio, barley, and rice into a variety of porridges, gruels, flatbreads, and beverages, enhancing shelf life, sensory quality, nutritional value, and microbiological safety [1]. Apart from their role as staple foods, these fermented cereal products represent an important factor of indigenous knowledge systems and continue to contribute to household nutrition and sustainable food systems across the region [2].

The diversity of traditional fermented cereals in SSA is reflected in several culturally important products, including *ogi*, *mahewu*, *togwa*, *kenkey*, *injera*, and *ting* [2]. *Ogi*, a fermented maize-based pap widely consumed in West Africa, particularly Nigeria, is produced through spontaneous fermentation involving diverse lactic acid bacteria (LAB) and yeasts and is traditionally consumed as a staple or complementary food [3]. *Mahewu* (*amahewu*), a non-alcoholic fermented cereal beverage widely consumed in Southern Africa, is commonly prepared from maize and/or other cereals with malted cereal flour and undergoes LAB- and yeast-associated fermentation [1]. Also, Pswarayi and Ganzle [1] reported that *Togwa*, traditionally consumed in Tanzania and neighbouring regions, is a fermented beverage or thin porridge produced from cereals such as maize, sorghum, and finger millet, with LAB and yeasts contributing to acidification and flavour development. In Ghana and other parts of West Africa, *kenkey* is produced from fermented maize dough and is characterised by substantial LAB activity during fermentation. In Ethiopia and Eritrea, *injera* is a sour fermented flatbread traditionally prepared predominantly from teff and is associated with complex communities of LAB and yeasts. Similarly, *ting*, a traditional sorghum-based fermented food consumed particularly in Botswana and neighbouring Southern African regions, undergoes LAB-dominated fermentation that contributes to acidification and the development of characteristic sensory and functional properties [2,3]. Collectively, these products reflect the geographical, cereal substrate, processing, and microbial diversity of traditional fermented cereals across SSA and provide important food matrices for investigating potential probiotic activity and microbiome-related health effects.

Cereal-based diets constitute a major source of daily energy intake in many SSA countries; however, reliance on cereal staples is often associated with limited protein quality and the presence of antinutritional compounds, particularly phytate, which reduce the bioavailability of essential minerals such as iron, zinc, and calcium. Fermentation partially overcomes these nutritional constraints through the activation of endogenous and microbial enzymes that hydrolyse macromolecules, reduce phytate, release bound phenolic compounds, improve protein digestibility, and enhance the accessibility of micronutrients and other bioactive constituents [4,5]. Consequently, fermentation has gained increasing consideration as a sustainable bioprocessing approach for improving the nutritional quality and functionality of cereal-based foods.

Traditional cereal fermentations are driven by complex microbial systems controlled by lactic acid bacteria (LAB) and yeasts, with commonly reported microorganisms including *Lactiplantibacillus plantarum*, *Limosilactobacillus fermentum*, *Levilactobacillus brevis*, *Pediococcus pentosaceus*, *Weissella* spp., *Leuconostoc* spp., *Lactococcus lactis*, and *Saccharomyces cerevisiae*. Cappozi et al. [6] and Franz et al. [3], reported that these microorganisms contribute to rapid acidification of the food matrix, inhibition of spoilage and pathogenic microorganisms, and production of numerous metabolites, including organic acids, exopolysaccharides, vitamins, γ-aminobutyric acid (GABA), bioactive peptides, and volatile flavour compounds that collectively improve food quality and functionality.

Interest in these microorganisms has increased due to their potential probiotic properties and potential influence on the human gut microbiota. Nevertheless, the detection of a microorganism in a fermented food does not necessarily imply probiotic properties. Hill et al. [7] stated that probiotics are viable microorganisms that, when consumed in adequate amounts, impart a health benefit on the host. Therefore, microorganisms isolated from traditional fermented cereals should not be classified as probiotics solely on the basis of their presence or in vitro functional characteristics; rather, those demonstrating relevant probiotic-associated properties may be described as having probiotic potential pending adequate evidence of safety, appropriate dosing, and a health benefit. Consequently, microorganisms identified solely through laboratory screening assays, including acid tolerance, bile tolerance, antimicrobial activity, or adhesion-related characteristics, are regarded as potential probiotic microorganisms rather than confirmed probiotics unless supported by appropriate strain-level characterisation and evidence of health benefits in humans.

Growing recognition of the central role of the gut microbiota in regulating nutrient metabolism, immune function, intestinal barrier integrity, and susceptibility to chronic disease has stimulated interest in fermented foods as dietary modulators of microbial ecology. Fermented cereal foods may influence the gut ecosystem through multiple mechanisms, including the delivery of viable microorganisms, fermentable dietary fibre, resistant starch, polyphenols, exopolysaccharides, and fermentation-derived metabolites [8]. These interactions may promote the production of short-chain fatty acids and other microbial metabolites associated with gastrointestinal, metabolic, and immune health. However, research on traditional fermented cereals in SSA has predominantly focused on fermentation microbiology, probiotic screening, nutritional composition, bioaccessibility, and laboratory-based functional properties, with comparatively limited investigation of human dietary responses, inter-individual variability, and microbiome stratification. This evidence gap is particularly relevant to precision nutrition, which seeks to account for differences in individual characteristics, including dietary, metabolic, and microbiome profiles, when developing nutrition strategies.

Although previous reviews have examined fermented foods, probiotics, fermented cereals, and the nutritional effects of fermentation, few have integrated these areas with gut microbiota modulation and microbiome-informed precision nutrition, particularly within the context of SSA. Existing reviews have also tended to emphasise food-level microbial characterisation or probiotic supplementation rather than considering fermented cereals as complex food matrices in which cereal-derived substrates, fermentation metabolites, and microbial communities may interact to influence biological responses. Furthermore, the available literature has rarely been systematically mapped across study designs, distinguishing mechanistic evidence from findings supported by human studies. This distinction is important because evidence derived from in vitro and animal models cannot necessarily be extrapolated to human health outcomes or personalised dietary responses.

Accordingly, this scoping review considers precision nutrition as an emerging translational framework rather than an established clinical application. The review maps the available evidence linking traditional fermented cereals with microbial and probiotic characteristics, fermentation-associated metabolites, gut microbiota modulation, and human health outcomes, while identifying geographical, methodological, and translational gaps in the evidence base. By integrating evidence across in vitro, animal, observational, and human intervention studies, the review provides a structured evidence map of the current scientific foundation and identifies the research priorities required to determine whether traditional fermented cereals could contribute to future microbiome-informed personalised nutrition strategies in SSA.

Against this background, the scoping review aimed to map the available evidence linking traditional fermented cereals to probiotic functionality, gut microbiota modulation, and human health in SSA, and to assess the extent to which the existing evidence supports their potential relevance to microbiome-informed precision nutrition. Specifically, the review sought to (i) identify the traditional fermented cereal foods and cereal substrates investigated for probiotic and health-promoting properties; (ii) characterise the predominant microorganisms, particularly lactic acid bacteria and yeasts, together with their reported functional attributes; (iii) explore fermentation-induced bioactive metabolites and food-matrix modifications that may contribute to biological activity; (iv) synthesise the available evidence regarding gut microbiota modulation, microbial functionality, and short-chain fatty acid production; (v) evaluate the reported human health outcomes across in vitro, animal, observational, and intervention studies; and (vi) highlight the main geographical, methodological, and translational research gaps that need be addressed to enable the integration of traditional fermented cereals into microbiome-informed precision nutrition strategies in Sub-Saharan Africa.

## 2. Methods

### 2.1. Design and Reporting

This research followed a scoping review to systematically source, map, and synthesise the existing evidence on the probiotic nature of traditional fermented cereals, their impact on gut microbiota modulation, and their implications for human health and precision nutrition in Sub-Saharan Africa. It was appropriate that a scoping review methodology be applied as the topic area includes a wide and multidisciplinary literature, among food science, microbiology, nutrition, and health sciences, with diverse study designs and emerging concepts that have not been comprehensively synthesised.

The review was performed using Arksey and O’Malley’s [8] methodological framework, with their development model showing five stages of scoping reviews: (i) identifying the research question, (ii) identifying relevant studies, (iii) selecting eligible studies, (iv) charting the data, and (v) collating, summarising, and reporting the findings. The same refinements proposed by Levac et al. [9] were also integrated into the review to improve methodological rigour, which promotes precise review aims, an iterative process of study selection, consensus decision-making among reviewers, and analysis of findings rather than a purely descriptive representation. Moreover, the review followed the renewed methodological guidance for scoping reviews published by the Joanna Briggs Institute (JBI), which outlines key recommendations for developing eligibility criteria, conducting systematic searches, extracting information, and presenting evidence as transparently and reproducibly as possible [10].

The reporting of this review was guided by the Preferred Reporting Items for Systematic Reviews and Meta-Analyses Extension for Scoping Reviews (PRISMA-ScR), to improve the transparency, completeness, and reproducibility of scoping reviews [11]. Accordingly, the study selection process was presented using a PRISMA-ScR flow diagram illustrating the identification, screening, eligibility assessment, and inclusion of studies. Since the primary objective of a scoping review was to map the breadth and nature of the available evidence rather than to assess the effectiveness of interventions, no formal risk-of-bias assessment was undertaken, in accordance with current methodological recommendations for scoping reviews.

### 2.2. Eligibility Criteria

Eligibility criteria were structured using the Population–Concept–Context (PCC) framework. Human participants of any age were eligible. Animal and in vitro studies were also included when they directly investigated microorganisms, fermentation-derived metabolites, gut microbiota interactions, or health-related mechanisms associated with traditional fermented cereals from SSA. The concept encompassed traditional fermented cereal foods, their microbial ecology, probiotic-related characteristics, fermentation-derived metabolites, gut microbiota outcomes, and health-related outcomes. The context included Sub-Saharan Africa (SSA) or traditional fermented cereal products originating from SSA but investigated elsewhere. Peer-reviewed original research articles published in English were eligible for inclusion. Editorials, conference abstracts, dissertations, reviews, commentaries, and studies focusing exclusively on non-cereal fermented foods were excluded.

### 2.3. Information Sources, Search, and Strategy

Literature searches were conducted in Scopus, PubMed, Web of Science, ScienceDirect, and Google Scholar using structured search strategies adapted to the indexing characteristics of each database. Searches covered the period from 2000 to 2026. The search strategy combined terms relating to traditional fermented cereals and named products, microorganisms and probiotic characteristics, gut microbiota and microbiome-related outcomes, health effects, and Sub-Saharan Africa. The term precision nutrition was not required as a mandatory search concept because it represents the translational framework through which the evidence was interpreted, and its inclusion as a compulsory search term could have excluded relevant studies investigating microbiota, metabolic, nutritional, or health outcomes without explicitly using the term precision nutrition.

For Scopus, the search strategy included combinations of terms such as “traditional fermented cereal”, *ogi*, *mahewu*, *togwa*, *kenkey*, *injera*, *ting*, probiotic, “lactic acid bacteria”, yeast, “gut microbiota”, microbiome, metabolite, health, and “Sub-Saharan Africa” or Africa. The PubMed strategy combined relevant Medical Subject Headings (MeSH) terms with free-text keywords, while equivalent combinations of keywords were used in Web of Science, ScienceDirect, and Google Scholar.

### 2.4. Selection of Evidence Sources

**Figure 1:**
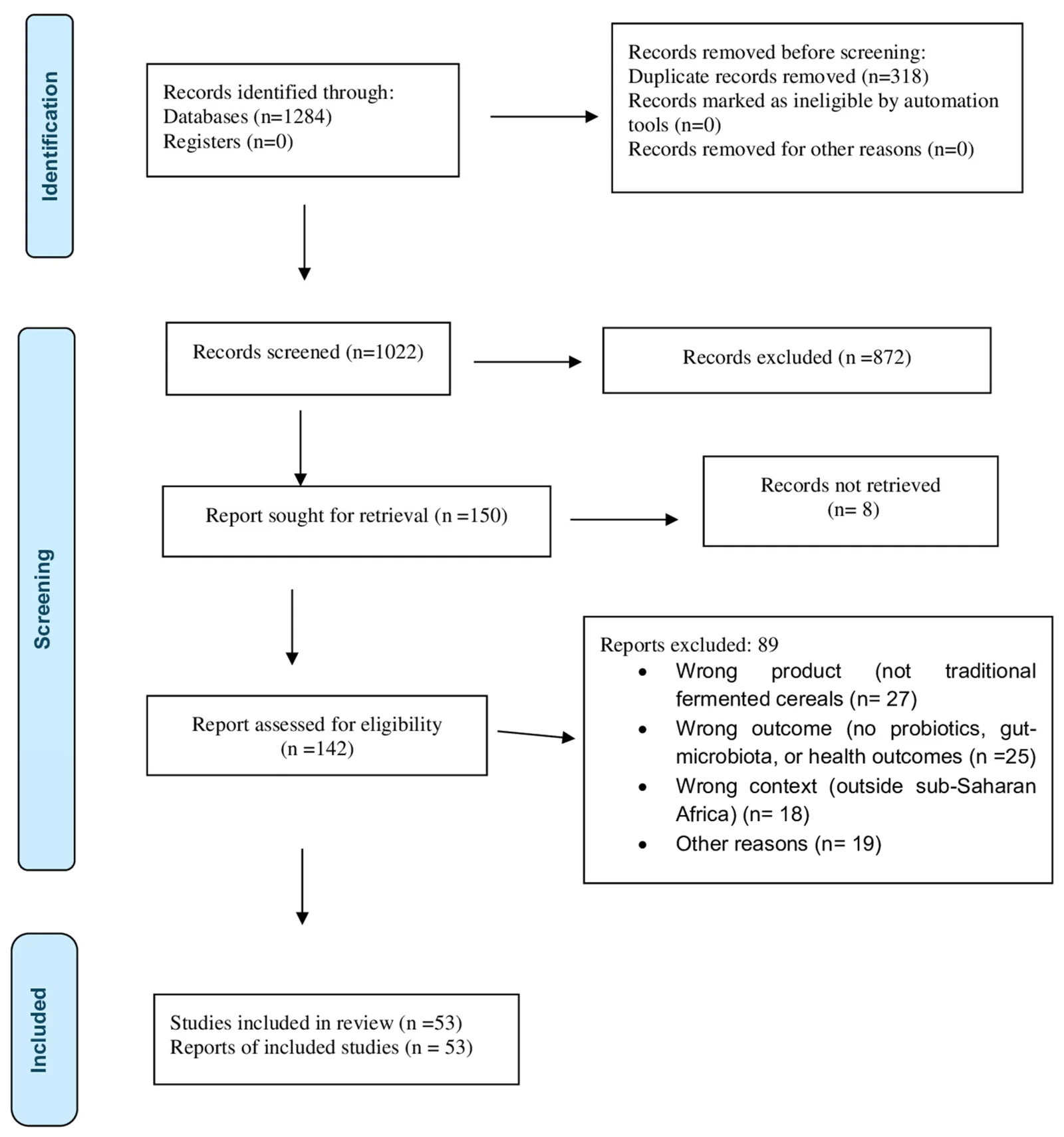
PRISMA-ScR flow diagram for study identification and selection.

All records retrieved from the electronic database searches were imported into EndNote 20 (Clarivate Analytics, Philadelphia, PA, USA) for reference management and duplicate removal. Following deduplication, the remaining records were exported for the study selection process. The study selection process is presented in Figure 1 in accordance with the PRISMA-ScR guidelines.

Two reviewers independently screened the titles and abstracts of all retrieved records against the predefined eligibility criteria. Studies considered potentially relevant were subsequently subjected to full-text assessment to determine their eligibility for inclusion in the review. Full-text articles were independently evaluated by the same reviewers using the established inclusion and exclusion criteria. Any disagreements arising during the screening or full-text assessment were resolved through discussion and consensus. The reasons for excluding studies at the full-text screening stage were systematically documented to ensure transparency and reproducibility of the review process. The entire study selection procedure was conducted and reported in accordance with the Preferred Reporting Items for Systematic Reviews and Meta-Analyses Extension for Scoping Reviews (PRISMA-ScR) guidelines [11], and the PRISMA-ScR checklist form for the study was attached as Supplementary Material.

### 2.5. Data Charting

The extraction form captured the author, year, country, product, cereal substrate, fermentation method, study design, sample or population, microbial identification method, strains, probiotic assays, metabolites, gut microbiota outcomes, health outcomes, principal findings, and limitations. Two reviewers independently charted or verified the data.

### 2.6. Critical Appraisal

The primary objective of this scoping review was to map and synthesise the breadth, characteristics, and nature of the available evidence rather than to evaluate the effectiveness of interventions or estimate pooled effect sizes. Accordingly, a formal methodological quality assessment or risk-of-bias appraisal was not undertaken, which was consistent with the methodological guidance for scoping reviews developed by the Joanna Briggs Institute (JBI) and the PRISMA Extension for Scoping Reviews (PRISMA-ScR) [11]. This approach aligns with the purpose of scoping reviews, which is to identify knowledge gaps, summarise the available evidence, and clarify key concepts rather than exclude studies based on methodological quality.

Although a formal critical appraisal was not performed, the potential influence of study design and methodological characteristics on the interpretation of findings was considered throughout the synthesis. The included studies were categorised as in vitro, animal, observational human, and controlled human intervention studies. This classification facilitated interpretation of findings according to their respective levels of inference and helped prevent mechanistic and laboratory-based findings from being interpreted as definitive evidence of human health effects.

An evidence hierarchy was used to guide the interpretation of findings rather than to formally score or rank study quality. In vitro studies were interpreted primarily as evidence of biological plausibility and potential mechanisms, whereas animal studies were considered supportive of potential physiological effects but not directly generalisable to humans. Observational human studies were used to identify potential associations but were not considered sufficient to establish causal relationships. Greater interpretive weight was given to controlled human intervention studies, particularly randomised studies, because these provide stronger evidence for evaluating causal effects on human health outcomes. Thus, conclusions regarding probiotic functionality, gut microbiota modulation, and human health were drawn cautiously and in accordance with the level of evidence provided by each study design.

The evidence hierarchy adopted in this review is presented in Table 1 and illustrates the relative inferential contribution of each study design to understanding the relationships among traditional fermented cereals, microbial and probiotic characteristics, gut microbiota modulation, human health, and precision nutrition. Considerable methodological heterogeneity was observed across the included studies, including differences in study design, fermented cereal products, microbial strains, experimental conditions, outcome measures, and approaches to assessing gut microbiota and health-related effects. Rather than being treated as a basis for quantitative comparison, this heterogeneity was considered in the narrative synthesis and reinforced the scoping review approach’s suitability for mapping the evidence base.

Consequently, study quality was not formally scored, and no study was excluded on the basis of methodological quality. Instead, findings were interpreted according to the study design, methodological characteristics, and the predefined evidence hierarchy, with particular caution when translating in vitro and animal findings to human health and precision-nutrition applications.

### 2.7. Synthesis

The evidence was synthesised using descriptive and thematic approaches. Tables and figures were used to categorise the traditional fermented cereal products, cereal substrates, microorganisms, probiotic-related characteristics, fermentation-associated metabolites, gut microbiota outcomes, and reported health effects. Findings were organised according to study design and outcome category to distinguish mechanistic evidence from evidence derived from human studies. A meta-analysis was not undertaken due to substantial heterogeneity across fermented cereal products, fermentation processes, microbial strains, study populations or experimental models, exposure conditions, outcome measures, and methods used to assess gut microbiota and health-related effects. During the narrative synthesis, findings were interpreted in relation to their study designs and corresponding levels of inference, with particular caution when extrapolating findings from in vitro and animal studies to human health outcomes. Human observational and intervention findings were considered separately to distinguish associations from evidence of potential causal effects.

## 3. Results

The literature search identified 1284 records from electronic databases, including PubMed, Scopus, Web of Science, ScienceDirect, and Google Scholar, with an additional 56 records identified through other sources, including reference-list screening, citation searching, and manual searching. Following the removal of 318 duplicate records, 1022 records remained for title and abstract screening. Of these, 872 records were excluded because they did not meet the predefined eligibility criteria. Consequently, 150 full-text reports were sought for retrieval, of which 8 could not be retrieved. A total of 142 full-text reports were assessed for eligibility, and 53 studies met the predefined inclusion criteria and were included in the final scoping review.

The principal reasons for excluding the 89 full-text reports were wrong product (studies investigating products other than traditional fermented cereals; *n* = 27), wrong outcome (studies that did not evaluate probiotic-related characteristics, gut microbiota modulation, or relevant human health outcomes; *n* = 25), wrong context (studies conducted outside Sub-Saharan Africa without a relevant SSA-origin fermented cereal product; *n* = 18), and other reasons (*n* = 19), including review articles, conference abstracts, duplicate publications, and studies with insufficient information to address the review objectives. Finally, the 53 included studies formed the evidence base for mapping probiotic-related microorganisms and functionality, gut microbiota modulation, fermentation-associated outcomes, and human health effects associated with traditional fermented cereals in Sub-Saharan Africa.

### 3.1. Characteristics and Distribution of Included Studies

A total of 53 studies published between 2000 and 2026 met the predefined eligibility criteria and were included in this scoping review. Studies were conducted across several Sub-Saharan African countries, with the largest number reported from Nigeria, South Africa, Ethiopia, Ghana, and Tanzania. A smaller number of studies were identified from Central African and other Southern African countries, indicating an uneven geographical distribution of the available evidence.

A substantial proportion of the included studies used culture-dependent approaches for microbial isolation and phenotypic characterisation, with many assessing characteristics associated with probiotic potential, including antimicrobial activity, acid and bile tolerance, and fermentation-related properties. In contrast, relatively few studies employed advanced molecular and systems-level approaches, such as 16S rRNA amplicon sequencing, shotgun metagenomics, metabolomics, or whole-genome sequencing. Similarly, animal studies and controlled human intervention studies were less frequently represented in the evidence base. This distribution indicates important methodological gaps, particularly in the application of molecular microbiology, microbiome profiling, metabolomics, and human intervention approaches to traditional fermented cereals in SSA. This pattern is consistent with previous reviews of African fermented foods, which have reported a strong emphasis on food-level microbial characterisation and comparatively limited application of genome-resolved and microbiome-based approaches [2].

### 3.2. Traditional Fermented Cereal Foods

The 53 studies included in this scoping review encompassed a diverse range of traditional fermented cereal foods consumed across Sub-Saharan Africa. These products were predominantly made from maize, sorghum, pearl millet, finger millet, and teff, reflecting the region’s major staple cereals. The most frequently investigated products were *ogi* (*akamu*/pap) from West Africa, *mahewu* (*amahewu*) from Southern Africa, *togwa* and *uji* from East Africa, *kenkey* and *koko* from Ghana, *injera* from Ethiopia and Eritrea, *ben-saalga* from Burkina Faso, *kisra* from Sudan, and *munkoyo* from Zambia [2,25]. The major types of cereal substrates, processing methods, geographical distribution, and the relevance of evidence for these traditional fermented foods are shown in Table 2. 

Despite regional differences in ingredients and processing techniques, the included studies showed that many of these products relied on spontaneous or, in some cases, starter-mediated fermentation, with lactic acid bacteria (LAB) and yeasts frequently reported as key microbial groups [2,3]. The composition and succession of microorganisms varied according to the cereal substrate, geographical location, fermentation conditions, and processing practices [2,3]. The reported effects of fermentation included acidification, inhibition of undesirable microorganisms, improved digestibility, sensory development, and changes in nutritional characteristics [3,25].

Across the included evidence, a major research focus was the characterisation of LAB and yeast populations, their roles in fermentation, and the assessment of characteristics associated with probiotic potential. Studies also examined reductions in antinutritional factors, changes in mineral bioaccessibility, acidification, antimicrobial activity, and improvements in digestibility [2,25,26]. In contrast, relatively fewer studies investigated outcomes beyond food-level microbiology, including gut microbiota modulation, metabolomic changes, and human health outcomes. This distribution indicates that the available evidence remains weighted towards food microbiology and laboratory-based characterisation, with comparatively limited evidence addressing the gut microbiota and human health implications of traditional fermented cereals.

The types of fermented cereal products studied varied according to regional food traditions. Research from West Africa mainly examined fermented porridges, while studies from Southern Africa focused more on fermented beverages. In East Africa, both porridges and beverages received attention, whereas research from Ethiopia concentrated largely on fermented flatbreads.

### 3.3. Microbial Diversity and Probiotic Potential Functions

The studies included in this scoping review demonstrated that traditional fermented cereal foods in Sub-Saharan Africa harbour diverse microbial communities, predominantly comprising lactic acid bacteria (LAB) and yeasts (Table 3). The reported microbial communities exhibited patterns of succession during fermentation, with early microorganisms such as *Leuconostoc*, *Weissella*, *Lactococcus*, and *enterococci* occurring during the initial stages, followed by more acid-tolerant LAB as fermentation progressed. The composition and succession of microorganisms varied according to the cereal substrate, geographical location, fermentation conditions, and processing practices [2,3]. Yeasts, particularly *Saccharomyces cerevisiae*, as well as *Pichia* and *Wickerhamomyces* species, were also reported and may contribute to fermentation, flavour development, nutrient transformation, and interactions with LAB [3,31].

The microorganisms identified in the included studies were interpreted as microorganisms with putative probiotic potential rather than as clinically validated probiotics. Most studies assessed in vitro characteristics associated with probiotic potential, including acid tolerance, bile tolerance, antimicrobial activity, gastrointestinal survival, adhesion-related properties, and enzyme production. However, these laboratory-based characteristics alone do not establish probiotic status, because a probiotic requires evidence that a live microorganism, when administered in adequate amounts, confers a demonstrated health benefit on the host [7]. Accordingly, the probiotic-related findings were interpreted cautiously and at the strain level.

Across the reviewed studies, *Lactiplantibacillus plantarum* was frequently reported in traditional fermented cereal products, including *ogi*, *kenkey*, *mahewu*, *togwa*, and *injera*, as well as other sour cereal-based foods [2,3]. Reported characteristics of *L. plantarum* included rapid acidification, production of organic acids and antimicrobial compounds, and adaptation to acidic fermentation environments. However, these characteristics are strain-dependent and should not be interpreted as evidence that all *L. plantarum* isolates from fermented cereals possess probiotic activity [7,15].

*Limosilactobacillus fermentum* was also reported in maize, sorghum, and millet-based fermentations and investigated for characteristics such as antimicrobial activity and tolerance to simulated gastrointestinal conditions. These findings indicate probiotic potential that guarantees further strain-specific investigation, but in vitro evidence alone is insufficient to establish a health benefit in humans [7,16].

Other LAB reported in African cereal fermentations included *Levilactobacillus brevis*, *Pediococcus pentosaceus*, *Weissella* spp., *Leuconostoc* spp., and *Lactococcus lactis* [2,3]. Their reported technological and functional characteristics included acidification, exopolysaccharide production, flavour development, antimicrobial activity, and interactions with other members of the fermentation microbiota. Because these characteristics may vary substantially among strains, they should not be generalised across an entire species.

Yeasts constituted another important component of the fermentation microbiota. *Saccharomyces cerevisiae* was reported in fermented cereal products including *injera* and *togwa*, where it may contribute to fermentation, flavour development, carbon dioxide production, and interactions with LAB [3,31]. Other yeast genera, including *Pichia* and *Wickerhamomyces*, have also been detected in cereal fermentations. However, their technological, functional, and safety characteristics require product- and strain-specific investigation.

### 3.4. Gut–Microbiota Effects

Among the 53 studies included in this scoping review, relatively few directly evaluated changes in gut microbiota following consumption of traditional fermented cereal foods in human participants (Table 4). A substantial proportion of the available evidence was derived from in vitro fermentation experiments, laboratory-based characterisation of microorganisms, animal models, and other mechanistic investigations rather than controlled human intervention studies. Consequently, the evidence for gut–microbiota effects was predominantly indirect or mechanistic, with limited direct evidence from comprehensive analyses of the human gut microbiome.

The potential contributions of ingested lactic acid bacteria (LAB) and yeasts to host–microbiota interactions have been investigated and discussed in several studies. However, gastrointestinal survival or recovery of an ingested microorganism does not, by itself, demonstrate long-term persistence or sustained modification of the resident gut microbiota [15,46]. This distinction is important when interpreting studies that report probiotic-associated characteristics without directly measuring changes in the composition or function of the human gut microbiota.

Species including *Lactiplantibacillus plantarum*, *Limosilactobacillus fermentum*, *Levilactobacillus brevis*, and *Saccharomyces cerevisiae* were investigated for characteristics relevant to gastrointestinal survival and probiotic potential, including tolerance to simulated gastric and bile conditions, antimicrobial activity, and adhesion-associated properties. These findings indicate the potential of selected microorganisms to survive or interact with gastrointestinal environments; however, they do not establish long-term persistence in the gastrointestinal tract or clinically meaningful modification of the human gut microbiota [7,46].

Fermented cereal foods also contain fermentable dietary fibre, resistant starch, arabinoxylans, β-glucans, and other microbiota-accessible carbohydrates that may serve as substrates for intestinal microorganisms. The included evidence indicates that these substrates can influence microbial growth and metabolic activity and may support the production of short-chain fatty acids (SCFAs), including acetate, propionate, and butyrate, although the magnitude and direction of these effects depend on substrate characteristics and the composition and functional capacity of the resident microbiota [47,48]. SCFAs have established roles in intestinal epithelial function, energy metabolism, and immune regulation. However, direct evidence demonstrating that consumption of traditional fermented cereals from SSA produces sustained increases in SCFA concentrations in humans remains limited.

Fermentation-associated metabolites may also contribute to changes in the physicochemical and functional characteristics of cereal foods. Organic acids produced during fermentation can lower food pH and inhibit undesirable microorganisms, thereby improving food-level microbiological safety [2,15]. These antimicrobial effects within the food matrix should, however, be distinguished from evidence of direct or sustained modulation of the human gut microbiota. Fermentation may also increase the bioaccessibility of phenolic compounds by releasing compounds bound to cereal matrices. Polyphenols can interact bidirectionally with the gut microbiota, while microbial transformation of phenolic compounds can generate metabolites that may affect host physiology [50]. Nevertheless, evidence directly demonstrating that these interactions produce reproducible gut–microbiota or health effects following consumption of specific traditional fermented cereals in SSA remains insufficient.

Additional fermentation-derived compounds reported across the literature include exopolysaccharides, oligosaccharides, bioactive peptides, and γ-aminobutyric acid (GABA). LAB-derived exopolysaccharides have been investigated for potential prebiotic, technological, and host-interactive functions, although their effects depend on molecular structure, concentration, and strain-specific production characteristics [52,53]. Similarly, the biological relevance of fermentation-derived peptides, oligosaccharides, and GABA to gut–microbiota modulation requires further investigation using appropriate gastrointestinal, animal, and human models.

Overall, the available evidence supports biological plausibility for interactions among microorganisms associated with fermented cereals, food-derived substrates, fermentation-derived metabolites, and the intestinal ecosystem. However, direct evidence from controlled human studies demonstrating consistent and sustained changes in gut–microbiota composition, microbial function, or SCFA production following consumption of traditional fermented cereals in SSA remains limited. The current evidence, therefore, supports potential microbiota-related effects but is insufficient to establish consistent causal effects on the human gut microbiota or to support clinical application within precision-nutrition strategies.

### 3.5. Human-Health Outcomes

The evidence mapped in this scoping review identified a range of potential nutritional, microbiological, metabolic, and health-related effects associated with traditional fermented cereal foods, although the extent and strength of evidence varied considerably across outcomes (Table 5). Most of the evidence was derived from food chemistry, microbiological investigations, mechanistic studies, animal models, and observational research, whereas direct evidence from well-designed randomised controlled human interventions was limited. Accordingly, the health-related findings were interpreted according to study design and level of inference and should not be regarded as established clinical benefits in the absence of appropriate human evidence.

One of the most frequently investigated nutritional effects of cereal fermentation was improved digestibility and nutrient bioaccessibility. Fermentation may improve cereal digestibility through the partial hydrolysis of macromolecules and the reduction in antinutritional factors such as phytate. Fermentation and germination can activate endogenous and microbial enzymes that modify cereal carbohydrates, proteins, and antinutritional factors, potentially improving nutritional quality and nutrient bioaccessibility [5]. These biochemical changes may also influence mineral availability and carbohydrate digestion, although the magnitude of these effects varies according to the cereal substrate, fermentation conditions, and processing practices.

Fermentation may also contribute to microbiological safety through the production of organic acids and other antimicrobial compounds by lactic acid bacteria (LAB). These metabolites can reduce the pH of fermented cereal matrices and inhibit selected spoilage and pathogenic microorganisms. However, inhibition of microorganisms within the food matrix should not be interpreted as direct evidence of reduced diarrhoeal disease or improved gastrointestinal health in humans, particularly in the absence of controlled clinical studies.

The effects of fermentation on carbohydrate digestion and glycemic responses were also identified as areas of interest. Fermentation can modify starch structure, organic acid concentrations, and the physical characteristics of the cereal matrix, potentially altering the rate and extent of carbohydrate digestion. Dietary fibre, resistant starch, and fermentation-induced structural changes may influence postprandial glycemic responses; however, these effects depend on cereal type, processing conditions, formulation, and serving characteristics [47,54]. The available evidence therefore suggests that metabolic responses to fermented cereals are product- and context-dependent rather than a universal consequence of fermentation.

Fermented cereals may also interact with intestinal and immune functions through their microorganisms and fermentation-derived metabolites. Microorganisms and metabolites associated with fermentation have been proposed to influence immune signalling and intestinal barrier function, with supporting mechanisms reported in the broader probiotic literature [15,16]. However, direct evidence from interventions involving traditional fermented cereals in SSA remains limited. These effects should therefore be regarded as plausible biological pathways rather than established clinical effects on immune or intestinal health.

Potential cardiovascular effects represent another area of investigation, although the available evidence has been predominantly mechanistic. Proposed mechanisms include bile-salt hydrolase activity and the production of bioactive peptides with angiotensin-converting enzyme (ACE)-inhibitory activity. However, these mechanisms have mainly been examined through laboratory and nutritional studies, whereas direct evaluation of cardiovascular clinical outcomes following consumption of traditional fermented cereals in SSA remains limited. Accordingly, these findings indicate potential biological mechanisms but do not establish cardiovascular benefits in humans.

Similarly, fermentation may modify antioxidant and anti-inflammatory properties through changes in phenolic compounds and increased release or bioaccessibility of certain antioxidant constituents. Studies of fermented plant foods have reported changes in antioxidant activity following fermentation [55].

Nevertheless, antioxidant capacity measured chemically or in vitro does not necessarily translate into clinically meaningful antioxidant or anti-inflammatory effects in humans. The available evidence should therefore be interpreted cautiously until supported by appropriate human intervention studies.

**Figure 2:**
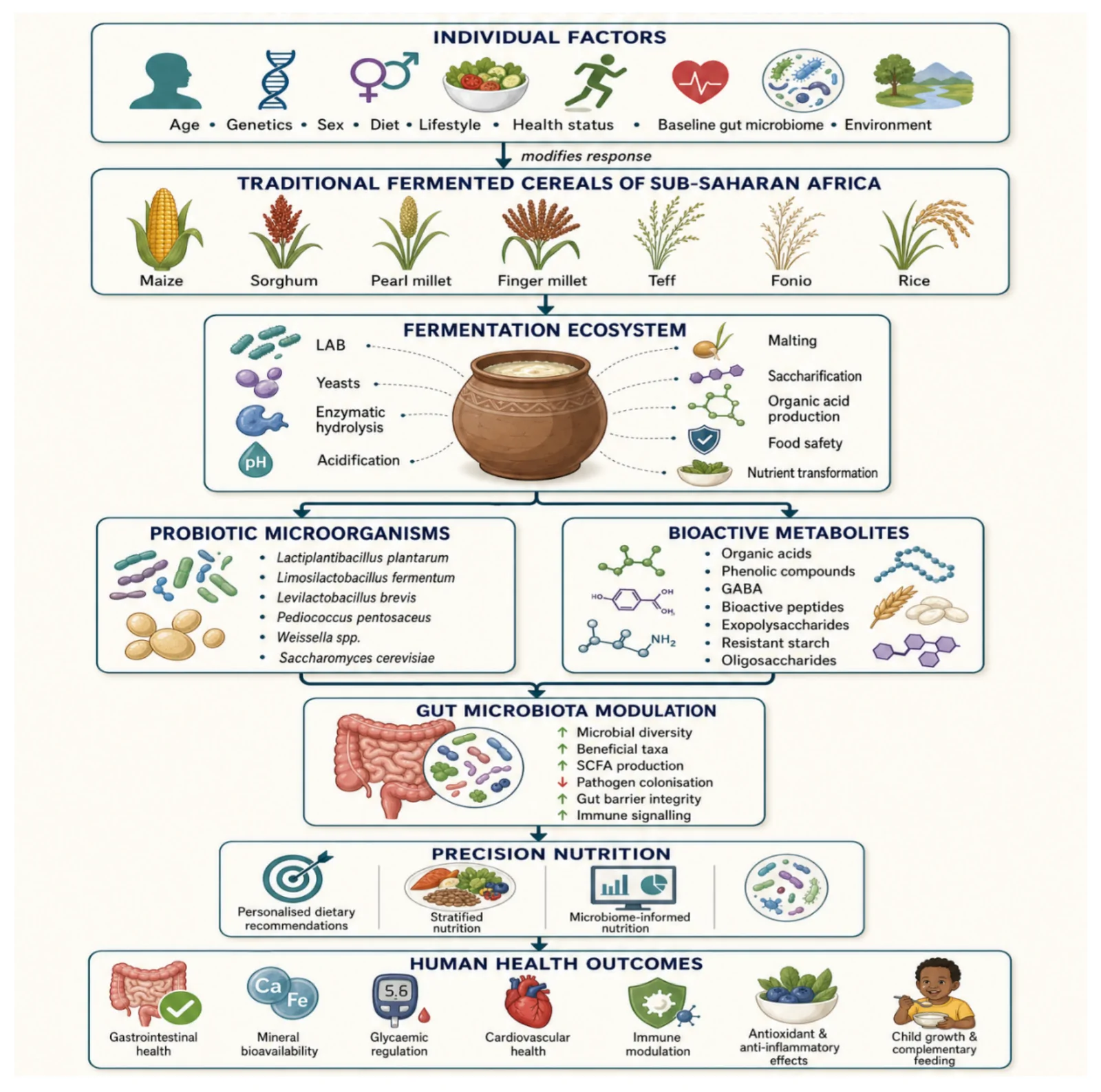
Conceptual framework linking fermented cereal matrices, fermentation ecology, probiotic activity, gut microbiota modulation, precision nutrition, and human-health outcomes.

The nutritional and functional characteristics of fermented cereals may have particular relevance to growth, complementary feeding, and food security in SSA. Traditional fermented cereal porridges may contribute to complementary feeding because fermentation can modify digestibility, acidity, sensory characteristics, and selected nutritional attributes. African cereal fermentations have been recognised as potentially useful for improving the nutritional quality and acceptability of cereal-based foods, particularly in resource-constrained settings [25,56]. However, their contribution to child nutrition and food security depends on adequate nutrient density, appropriate formulation, hygienic processing, and safe storage. Fermentation should therefore be regarded as a supportive food-processing strategy rather than a standalone solution to nutritional inadequacy or food insecurity. The relationships among the fermented cereal matrix, fermentation-associated microorganisms and metabolites, potential probiotic activity, gut–microbiota interactions, precision nutrition, and human-health outcomes are summarised in Figure 2.

Overall, the evidence map indicates that traditional fermented cereals have promising nutritional, microbiological, and functional characteristics, with potential implications for gastrointestinal, metabolic, immune, and nutritional outcomes. However, the strength of evidence varied substantially across outcomes, and much of the available evidence originated from laboratory, mechanistic, animal, or observational studies. A substantial gap, therefore, remains between biological plausibility and confirmation of clinically meaningful effects in humans. Further well-designed human intervention studies are required to establish whether the observed food-level and mechanistic effects translate into consistent improvements in gut microbiota composition or function, nutrient utilisation, metabolic health, immune responses, and other human-health outcomes.

## 4. Discussion

The evidence mapped in this scoping review supports the interpretation of traditional fermented cereals as biologically active food matrices rather than merely carriers of individual microorganisms. Fermentation can modify starch, proteins, cell-wall polysaccharides, phytate, and phenolic compounds while generating microbial biomass and fermentation-derived metabolites. These transformations have been documented in studies of cereal fermentation and African fermented foods, providing a mechanistic basis for considering fermented cereals as complex food–microbiome interfaces [2,3,25]. This whole-matrix perspective is important because the biological effects associated with fermented cereals may arise from interactions among microorganisms, fermentation-derived metabolites, and cereal-derived substrates rather than from the activity of individual microbial strains.

Nevertheless, the interpretation of microorganisms detected in fermented cereals requires caution, particularly regarding the use of the term “probiotic”. A recurrent limitation identified across the evidence base was the characterisation of isolates using in vitro acid-tolerance, bile-tolerance, antimicrobial, adhesion, or related assays without subsequent demonstration of a health benefit in humans. Accordingly, microorganisms identified primarily through laboratory screening should be described as having putative or potential probiotic properties rather than as established probiotics unless appropriate strain-level identity, safety, administration conditions, and a demonstrated health benefit are available. Future investigations should therefore incorporate genome-resolved strain identification, antimicrobial-resistance and virulence assessment, determination of viable cell numbers throughout storage, confirmation of survival in the finished food, and appropriately designed studies evaluating health effects.

This distinction between probiotic potential and demonstrated probiotic efficacy is particularly important when interpreting possible effects on the human gut microbiota. The evidence mapped in this review showed substantially more research on the microorganisms present in fermented foods than on changes in consumers’ intestinal microbiomes. The detection or ingestion of a potentially beneficial microorganism does not necessarily indicate persistent gastrointestinal establishment or sustained alteration of the resident gut microbiota [46]. Similarly, in vitro evidence of gastrointestinal tolerance, antimicrobial activity, or adhesion cannot be directly extrapolated to clinically meaningful modulation of the human microbiota. Direct translation will require controlled human feeding studies that combine detailed characterisation of the fermented food with longitudinal assessment of gut–microbiota composition and function, microbial metabolites, and relevant health outcomes.

The limited direct evidence for gut–microbiota effects also has important implications for interpreting the effects of traditional fermented cereals within a precision-nutrition framework. Fermented cereals may be relevant to precision nutrition because responses to dietary exposures can vary according to factors such as baseline microbiota composition, habitual dietary intake, age, nutritional status, host genetics, and health phenotype. However, the evidence identified in this review provides limited direct support for response-stratified effects of traditional fermented cereals in SSA. Precision nutrition should therefore be regarded as an emerging translational opportunity rather than an established clinical application in this context. Future studies should investigate whether baseline microbiota profiles and relevant host characteristics predict differential responses to specific fermented cereal products and whether such responses are reproducible across populations and dietary contexts.

The potential biological effects of fermented cereals should also be considered in the context of the whole food matrix. Traditional fermented cereals provide microorganisms alongside dietary fibre, resistant starch, phenolic compounds, organic acids, peptides, and other potentially microbiota-accessible substrates [15,47]. Consequently, observed biological responses should not necessarily be attributed to a single microbial strain. Future research should instead examine the combined contribution of microbial communities, cereal-derived substrates, and fermentation-derived metabolites, ideally using integrated microbiome, metabolome, and food-composition analyses. Such approaches could help determine whether specific combinations of microorganisms and food-derived substrates are associated with reproducible biological responses.

The whole-matrix perspective also has implications for standardisation and translation of traditional fermented cereals. Standardisation should not seek to eliminate the microbial and processing diversity that characterises traditional products. Rather, a balanced approach should document indigenous processing practices, identify functional microbial communities, establish appropriate safety and quality specifications, and investigate controlled fermentation or starter-culture approaches where these improve reproducibility without compromising desirable sensory and cultural characteristics. The use of appropriate starter cultures has been proposed as a strategy for improving the reproducibility and safety of small-scale fermentation systems [45]. Such approaches may help reduce batch-to-batch variability while retaining culturally relevant processing practices and product characteristics.

Beyond scientific and technological considerations, the potential applications of traditional fermented cereals should be considered within the broader public health, nutrition, and food system context of SSA. Fermented cereals may be particularly relevant in settings where access to refrigeration, diverse nutrient-dense foods, and advanced food-processing infrastructure is constrained. However, fermentation alone cannot compensate for poor raw-material quality, mycotoxin contamination, inadequate nutrient density, or poor post-fermentation hygiene [25]. Therefore, future translation of fermented cereals into nutrition or microbiome-informed interventions should integrate microbial and nutritional functionality with food safety requirements, quality control, affordability, accessibility, cultural acceptability, and sustainability.

Overall, the findings indicate that traditional fermented cereals represent promising food matrices through which microorganisms, dietary substrates, and fermentation-derived metabolites may interact with the gastrointestinal ecosystem. However, the current evidence remains stronger for fermentation-related biochemical and microbial changes than for reproducible gut microbiota effects or clinically meaningful human health effects. The principal research priority is therefore to move from descriptive and mechanistic studies towards well-characterised, adequately controlled human interventions capable of linking specific fermented cereal products and microbial or metabolic features with measurable and reproducible health outcomes. Establishing these relationships will be essential before traditional fermented cereals can be confidently incorporated into microbiome-informed precision-nutrition strategies in SSA.

### 4.1. Implications for Precision Nutrition

The findings of this review indicate that precision nutrition should currently be regarded as a future translational objective rather than an established application of traditional fermented cereals in Sub-Saharan Africa. The available evidence provides a biological foundation through the characterisation of fermentation-associated microorganisms, probiotic-related properties, gut–microbiota interactions, and fermentation-derived metabolites. However, direct evidence demonstrating that individual characteristics, such as baseline microbiota composition or host phenotype, predict differential responses to specific traditional fermented cereals remains limited. Consequently, the current evidence does not support the development of personalised dietary recommendations based on traditional fermented cereals.

The potential relevance of traditional fermented cereals to precision nutrition lies in their complex food matrix, which provides microorganisms, dietary substrates, and fermentation-derived metabolites that may interact with the gastrointestinal ecosystem. However, these interactions are likely influenced by interindividual factors, including baseline microbiota composition, habitual dietary intake, nutritional status, age, metabolic phenotype, and other host characteristics. Understanding this variability will be essential for determining whether specific fermented cereal products or fermentation-associated microbial communities can produce reproducible benefits in defined populations or responder groups.

Future research should therefore move beyond descriptive characterisation and isolated in vitro probiotic assays towards well-designed human intervention studies incorporating detailed food characterisation, strain-resolved microbiology, dietary assessment, host phenotyping, longitudinal microbiome profiling, and metabolomics. Where appropriate, integrated multi-omics approaches could help identify microbial and metabolic signatures associated with response to fermented cereal consumption. Adequately powered randomised controlled trials should then determine whether these biological signatures are associated with reproducible changes in gut–microbiota composition or function and clinically relevant health outcomes.

Accordingly, the translational implication of this scoping review is not that traditional fermented cereals are ready for immediate clinical application, but that they represent promising food matrices for future microbiome-informed dietary research. Establishing causal relationships between fermented cereal characteristics, individual biological profiles, microbiome responses, and health outcomes will be necessary before evidence-based precision-nutrition strategies can be developed and evaluated for populations in SSA.

### 4.2. Research Priorities

The study identified substantial progress in characterising the microbiology, nutritional properties, and functional potential of traditional fermented cereal foods in Sub-Saharan Africa, as contained in Table 6. Nevertheless, important scientific and translational gaps remain before these foods can be confidently integrated into evidence-based precision nutrition strategies. The current evidence is dominated by culture-dependent microbiology, food chemistry, and mechanistic studies, whereas high-quality human intervention trials, strain-level genomic characterisation, and integrated multi-omics investigations remain scarce. This pattern is consistent with previous reviews of African fermented foods, which have highlighted the need to complement descriptive microbial characterisation with approaches that provide greater insight into microbial functions and their interactions with the food matrix and host [2,27]. Addressing these deficiencies will improve the reliability, reproducibility, and clinical relevance of future research while facilitating the development of microbiota-directed dietary interventions tailored to African populations.

A key research priority is to strengthen evidence from human intervention studies. Although many laboratory and animal studies have demonstrated the probiotic potential and health-promoting properties of traditional fermented cereals, relatively few randomised controlled feeding trials have evaluated their effects in humans. The evidence mapped in this review was characterised by a predominance of in vitro and preclinical investigations, with limited direct evidence from controlled human feeding studies. Future studies should examine culturally relevant products prepared using traditional fermentation methods and consumed in realistic portions. Their effects on gut microbiota composition, microbial metabolites, nutritional biomarkers, and clinically important health outcomes should also be assessed. Such research is needed to determine whether consuming fermented cereals directly improves gastrointestinal health, metabolic regulation, immune function, and nutritional status.

Another important research priority is validating probiotic microorganisms at the strain level. Most studies identified microorganisms only at the species level, often using phenotypic or limited molecular methods. However, probiotic properties can vary considerably between strains of the same species. Future studies should therefore use whole-genome sequencing alongside thorough safety assessments, antimicrobial resistance and virulence screening, dose optimisation, and shelf-life testing. These measures are needed to confirm the identity, safety, stability, and probiotic functions of individual strains.

These approaches will help prevent exaggerated probiotic claims while supporting the development of well-characterised starter cultures that meet the requirements for industrial use and regulatory approval.

The study also emphasises the urgent need to move beyond simply describing microbial communities and focus more on understanding their functions within the gut microbiome. Most available studies have relied on culture-based methods or 16S rRNA sequencing, which primarily describe microbial composition rather than elucidate biological activity. Integrating shotgun metagenomics, meta-transcriptomics, fecal metabolomics, meta-proteomics, and host biomarker analyses would enable researchers to identify microbial genes, metabolic pathways, and functional interactions responsible for the observed health effects. Such multi-omics approaches would substantially strengthen mechanistic understanding of cereal–microbiome–host interactions.

Greater attention should also be given to standardising the food matrix and fermentation process. Current studies differ considerably in the cereal varieties used, fermentation conditions, starter cultures, back-slopping methods, fermentation time, temperature, pH, and viable microbial counts. Future research should follow standardised reporting guidelines that clearly describe these processing variables alongside microbiological and nutritional outcomes. Greater consistency in study methods and reporting would improve reproducibility, support future meta-analyses, and allow more reliable comparisons across products, laboratories, and geographical regions.

Future studies should also consider that individuals may respond differently to fermented foods. Factors such as baseline gut microbiota composition, age, sex, usual dietary habits, metabolic characteristics, genetics, and health status should be identified during the study design stage and included in the statistical analysis. Distinguishing between individuals who respond to an intervention and those who do not could support the development of more targeted dietary recommendations in line with precision nutrition.

The geographical distribution of the available evidence is also uneven, with most studies conducted in a small number of countries, particularly Nigeria, South Africa, Ghana, Ethiopia, Tanzania, and Kenya. Previous reviews of African fermented foods have similarly highlighted geographical and methodological gaps in the evidence base [27]. Future research should therefore include underrepresented areas, especially Central Africa and several Southern African countries. Greater attention should also be given to locally important fermented cereal products that remain poorly documented. Expanding research across the region would provide a better understanding of microbial diversity, processing practices, and cultural differences throughout Sub-Saharan Africa.

Finally, translating fermented cereal foods into effective public health interventions will require greater attention to food safety and practical implementation. Future studies should assess microbial safety, pathogen survival, mycotoxin contamination, antimicrobial resistance genes, consumer acceptance, production costs, shelf life, and the potential for large-scale production within local food systems. These considerations are particularly important because evidence of nutritional or microbiological functionality does not, by itself, establish that a fermented cereal product is safe, scalable, or suitable for population-level intervention.

## 5. Strengths and Limitations

**Figure 3:**
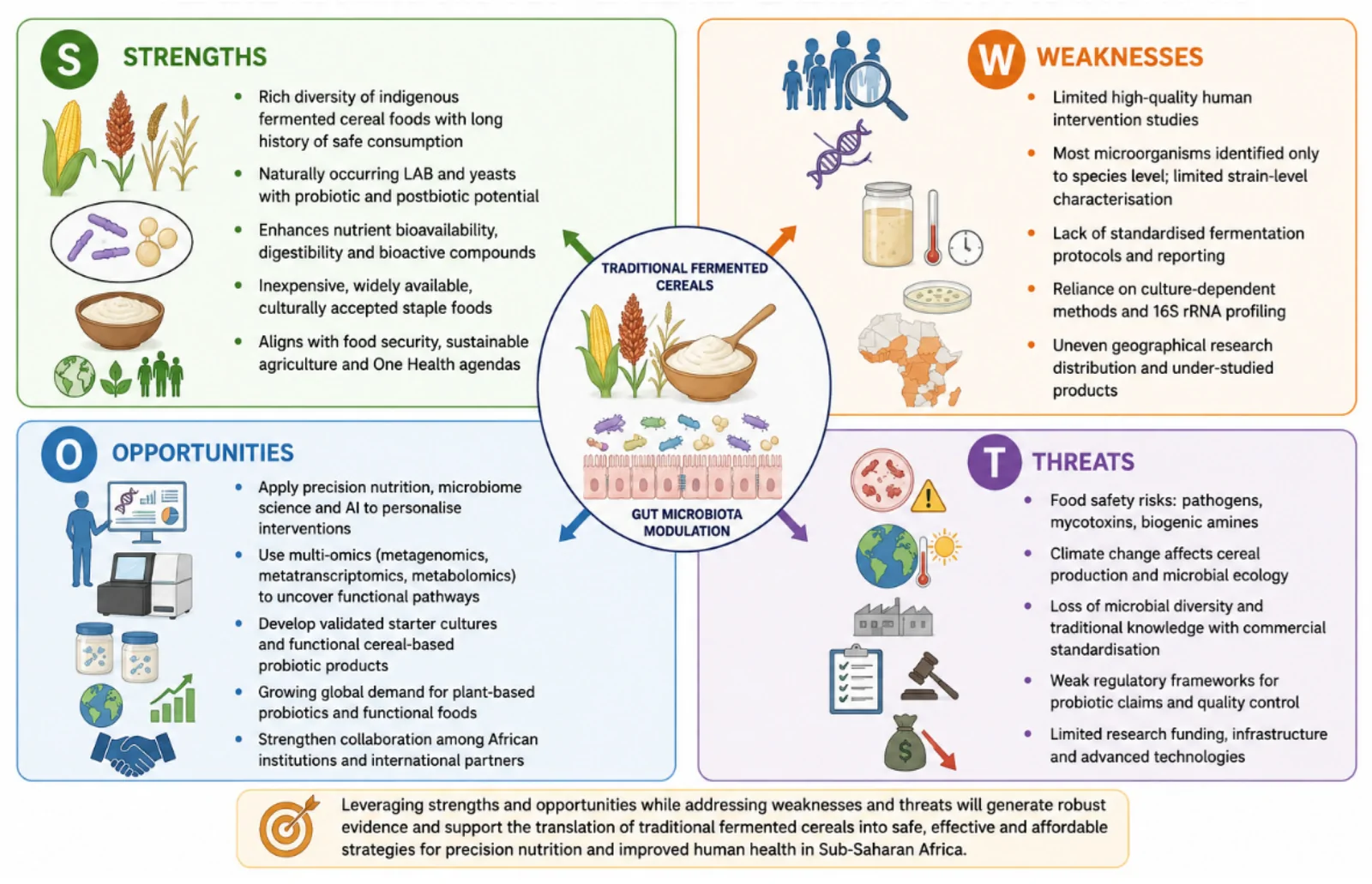
SWOT analysis of traditional fermented cereals for precision nutrition and gut microbiota modulation in sub-Saharan Africa.

This study brings together the available evidence on the connections between traditional fermented cereals, probiotic microorganisms, gut microbiota modulation, and precision nutrition in Sub-Saharan Africa (SSA). A major strength of this study is its integration of evidence across microbiology, nutrition, gut microbiota research, and precision nutrition, while systematically distinguishing established findings from emerging translational concepts. This broad evidence-mapping approach provides a more comprehensive perspective than previous narrative reviews focused on individual aspects of cereal fermentation. By drawing on research from food microbiology, food chemistry, nutrition, microbiome science, and public health, it highlights the potential of indigenous fermented cereal foods to support sustainable nutrition and better health. The SWOT analysis presented in Figure 3 further illustrates their key strengths, including diverse microbial communities, a long history of consumption, improved nutrient bioavailability, production of beneficial bioactive compounds, affordability, cultural acceptance, and relevance to food security and One Health goals.

Despite this potential, several limitations and research gaps remain. Much of the available research has been conducted in laboratories or has focused on the characteristics of the food products themselves. Relatively few well-designed human intervention studies have examined changes in the gut microbiota or clinically relevant health outcomes. Microorganisms are also commonly identified only at the species level, while genomic methods capable of distinguishing individual strains are rarely used. In addition, differences in cereal varieties, starter cultures, fermentation methods, processing conditions, and product names make it difficult to compare findings across studies. Many of the reported health benefits are based on mechanistic, in vitro, animal, or observational evidence and should therefore not be regarded as proof of clinical effectiveness.

The interpretation of the evidence is also constrained by the uneven geographical distribution of the available literature. Most included studies originated from a relatively small number of SSA countries, while several countries and traditional fermented cereal products remain poorly represented. This distribution may reflect differences in research capacity, funding, infrastructure, and publication activity rather than the actual distribution of fermented cereal practices across the region. The findings should therefore be interpreted as reflecting the current published evidence rather than as a complete representation of fermented cereal systems throughout SSA. Additional research from underrepresented countries and populations is needed to improve the geographical coverage and generalisability of future evidence.

The review process also involved several methodological limitations. Only peer-reviewed publications available in English were included, which may have introduced language and publication bias and resulted in the omission of relevant evidence published in other languages or in the grey literature. The exclusion of conference proceedings, dissertations, government reports, and technical documents may have further limited the breadth of the evidence identified. In addition, substantial heterogeneity was observed across cereal substrates, fermentation practices, microbial identification methods, analytical techniques, study populations, and outcome measures, limiting direct comparison between studies and precluding quantitative synthesis.

Furthermore, no formal risk-of-bias or evidence-certainty assessment was undertaken, consistent with the primary objective of a scoping review to map the extent, nature, and characteristics of available evidence rather than to estimate intervention effectiveness or determine certainty of effects. Accordingly, the findings should be interpreted as an evidence map rather than as confirmation of causal relationships or clinical efficacy.

To improve interpretability, the evidence was synthesised according to a predefined hierarchy of study designs, distinguishing mechanistic, preclinical, observational, and human intervention evidence. This approach allowed the strength of the available evidence to be considered when interpreting conclusions and reduced the risk of giving equivalent weight to laboratory findings and clinically derived evidence.

Despite these limitations, this review provides a structured synthesis of the current evidence linking traditional fermented cereals, probiotic-related microorganisms, gut microbiota modulation, and microbiome-informed precision nutrition in SSA. The findings should therefore be viewed primarily as an evidence map that identifies established knowledge, evidence gaps, methodological limitations, and priorities for future research, rather than as evidence of established clinical effectiveness.

## 6. Conclusions

Traditional fermented cereals are affordable, culturally accepted, and nutritionally important foods with considerable biological and functional potential in Sub-Saharan Africa (SSA). Products such as *ogi*, *mahewu*, *togwa*, *kenkey*, *injera*, and *ting* represent important components of regional food systems and provide complex food matrices containing fermentation-associated microorganisms, dietary substrates, and bioactive metabolites. The evidence mapped in this review indicates that fermentation can improve food quality, microbiological safety, nutrient bioaccessibility, and functional properties, while several microorganisms isolated from these foods exhibit probiotic-related characteristics. However, the available evidence remains predominantly mechanistic, laboratory-based, or preclinical, and is insufficient to establish consistent effects on gut microbiota or clinically meaningful human health outcomes.

The review therefore supports the biological plausibility and research potential of traditional fermented cereals as microbiome-informed functional foods, but not their current use as clinically validated precision-nutrition interventions. In particular, probiotic-related properties identified through in vitro assays should be regarded as preliminary evidence of probiotic potential rather than confirmation of probiotic efficacy. Similarly, evidence of antimicrobial activity, gastrointestinal tolerance, fermentation-associated metabolite production, or changes in food composition should not be interpreted as evidence of sustained gut–microbiota modulation or established health benefits in humans. Strong strain-level characterisation, safety assessment, and well-designed human intervention studies are required to establish whether specific microorganisms or fermented cereal products provide reproducible health benefits.

Precision nutrition should consequently be regarded as an emerging translational research direction rather than an established clinical application of traditional fermented cereals in SSA. Future research should prioritise adequately powered randomised controlled feeding studies that integrate detailed characterisation of the fermented food matrix with dietary assessment, host phenotyping, longitudinal gut-microbiome profiling, metabolomics, and, where appropriate, other multi-omics approaches. Such studies should also investigate inter-individual variability in response, including differences in baseline microbiota composition, habitual diet, metabolic phenotype, age, and other relevant host characteristics, to determine whether specific responder groups can be identified.

The review also identified important methodological and geographical gaps. Greater standardisation of fermentation conditions, microbial identification, safety assessment, and outcome reporting would improve comparability and reproducibility across studies. Expanding research to underrepresented countries and inadequately documented traditional fermented cereal products would strengthen the regional representativeness of the evidence base. Overall, the findings provide an evidence map of the current scientific landscape and identify a clear pathway for future research, rather than demonstrating clinical efficacy or readiness for immediate implementation of precision nutrition.

Realising the potential of traditional fermented cereals will require multidisciplinary collaboration among food scientists, microbiologists, nutritionists, clinicians, epidemiologists, and policymakers to generate robust human evidence, strengthen methodological consistency, and ensure that any future applications are safe, culturally appropriate, affordable, and supported by evidence. Such efforts could facilitate the responsible translation of indigenous fermented cereal foods into evidence-based nutrition and public-health strategies in SSA.

## Figures and Tables

**Table 1:** Evidence hierarchy used for the interpretation of included studies.

Study Design	Purpose	Strength of Evidence	Major Limitations	Interpretation	Reference
In vitro studies	Assess microbial characteristics, probiotic potential, fermentation metabolites, acid and bile tolerance, and antimicrobial activity	Low	Does not reflect the complexity of the human gastrointestinal tract	Used to identify potential probiotic candidates and biological mechanisms	[7,12,13,14]
Animal studies	Investigate physiological responses and gut microbiota modulation under controlled conditions	Moderate	Species differences limit direct extrapolation to humans	Provides mechanistic evidence supporting potential health effects	[15,16,17]
Observational human studies	Examine associations between fermented cereal consumption and health outcomes	Moderate	Confounding factors and inability to establish causality	Used to identify potential relationships requiring further investigation	[18,19,20]
Human intervention studies	Evaluate the causal effects of fermented cereals on gut microbiota and health outcomes	High	Sample sizes may be small; product formulations and probiotic strains may vary	Considered the strongest primary evidence for health-related conclusions	[12,21,22]
Systematic reviews and meta-analyses	Summarise and synthesise existing evidence from multiple primary studies	Very strong (secondary evidence)	Dependent on the quality, heterogeneity, and risk of bias of the included primary studies	Used to contextualise and compare findings rather than as primary evidence	[12,23,24]

**Table 2:** Traditional Fermented Cereal Foods.

Product	Principal Location	Cereal Substrate	Typical Processing	Evidence Relevance	Reference
*Ogi/Akamu/Pap*	Nigeria and neighbouring West Africa	Maize, sorghum or millet	Spontaneous soaking, wet milling and fermentation; cooked as porridge	LAB and yeasts; product microbiology, digestibility and complementary-feeding relevance	[2,26,27]
*Mahewu/Amahewu*	South Africa, Zimbabwe, Botswana, Namibia, Zambia	Maize or sorghum	Cooked cereal slurry followed by spontaneous or starter fermentation	Acidification, beverage safety, LAB diversity and non-alcoholic functional beverage development	[25,28,29,30]
*Togwa*	Tanzania and East Africa	Maize, sorghum, millet; sometimes cassava blends	Malting/saccharification followed by lactic fermentation	LAB–yeast ecology, antimicrobial activity and digestibility	[2,31,32]
*Kenkey*	Ghana	Maize	Dough fermentation followed by partial cooking and steaming	LAB succession, acidification, shelf life and staple-food quality	[2,3,33]
*Koko*	Ghana	Millet or maize	Spontaneous fermentation and cooking as porridge	Complementary-food use, phytate reduction, and microbial safety	[1,25,34]
*Injera*	Ethiopia and Eritrea	Teff, sorghum, barley, wheat, and maize	Multi-day sour fermentation and griddle baking	Mixed LAB–yeast fermentation, texture, nutrient bioaccessibility, and glycemic relevance	[3,25,35,36]
*Uji*	Kenya and East Africa	Maize, millet, and sorghum	Spontaneous fermented porridge	Food safety, starter-culture development, and child-feeding applications	[1,25,31,37]
*Ben-saalga*	Burkina Faso	Pearl millet	Wet processing, fermentation, and cooking	Complementary feeding, microbial ecology, and nutrient quality	[2,19,38]
*Kisra*	Sudan and neighbouring regions	Sorghum	Sour fermentation followed by thin-sheet baking	LAB/yeast ecology, digestibility, and staple-food quality	[2,3,39]
*Munkoyo*	Zambia and neighbouring countries	Maize or millet with plant-root amylase source	Saccharification and spontaneous fermentation	LAB/yeast fermentation, beverage quality, and indigenous processing	[1,3,27,40]

**Table 3:** Major Microorganisms Identified in Traditional Fermented Cereal Foods and Their Functional Attributes.

Microorganism/Group	Reported Products	Potential Functions	Interpretive Caution	Reference
*Lactiplantibacillus plantarum*	*Ogi*, *kenkey*, *mahewu*, *togwa*, *injera*, and other sour cereal fermentations	Rapid acidification; acid and bile tolerance; pathogen inhibition; enzyme production; phytate degradation; adhesion properties reported for selected strains	Detection at the species level alone does not confirm probiotic efficacy; strain-level identification and clinical validation are required.	[1,3,7,41]
*Limosilactobacillus fermentum*	Maize-, sorghum- and millet-based fermentations	Acidification, antimicrobial activity, oxidative stress-related properties and simulated gastrointestinal survival	Safety and clinical efficacy remain to be confirmed through vivo studies.	[3,16,31]
*Levilactobacillus brevis*	Sour porridges and fermented beverages	Heterolactic fermentation, flavour development, acid tolerance, and antimicrobial metabolite production	Functional characteristics vary considerably among strains.	[1,3,42]
*Pediococcus pentosaceus*	*Ogi*, millet and sorghum fermentations	Acid production, bacteriocin-like activity, and simulated gastrointestinal survival	Genomic safety evaluation is recommended before probiotic application.	[3,7,43]
*Weissella* spp.	Early stages of cereal fermentations	Exopolysaccharide production, texture improvement, and acidification	Taxonomic resolution and safety assessments are frequently incomplete.	[2,44]
*Leuconostoc mesenteroides*	Early maize and millet fermentations	Acidification, aroma development and exopolysaccharide production	Usually transient during fermentation and supported by limited probiotic evidence.	[2,45]
*Lactococcus lactis*	Selected cereal fermentations	Acidification and bacteriocin production	Food origin alone does not imply intestinal probiotic functionality.	[2,3,7]
*Saccharomyces cerevisiae*	*Injera*, cereal beers, *togwa* and mixed fermentations	Leavening, flavour formation, vitamin production and ecological interaction with LAB	Probiotic claims require defined, viable strains and supporting evidence in the host.	[1,15,16]
*Pichia/Wickerhamomyces* spp.	Mixed cereal fermentations	Aroma formation, phytase activity, enzymatic degradation and microbial interaction	Some isolates showed probiotic potential, but safety assessment is needed to establish probiotic status.	[2,31]

**Table 4:** Health outcomes associated with traditional fermented cereal foods.

Exposure	Microbiota Effect	Confirmatory Design	Evidence Limitation	Reference
LAB or yeast intake	Potential transient enrichment of ingested taxa and ecological interactions	Human intervention studies with strain-resolved sequencing	Limited direct SSA cereal evidence; long-term persistence should not be assumed	[15,46]
Fermentable fibre and resistant starch	Enrichment of saccharolytic taxa and increased acetate, propionate or butyrate	Faecal sequencing plus targeted SCFA analysis	Effects depend on cereal variety, processing and baseline microbiome	[47,48,49]
Organic acids and reduced food pH	Suppression of selected pathogens and altered substrate delivery to the colon	In vitro gut models, pathogen challenge and human studies	Food-level antimicrobial effects are not equivalent to durable microbiome modulation	[2,3,15]
Polyphenols and released phenolic acids	Selection of polyphenol-metabolising microbes and reciprocal production of bioactive metabolites	Metagenomics/metabolomics and controlled feeding	Bioaccessibility and dose frequently unreported	[47,49,50,51]
Exopolysaccharides and oligosaccharides	Potential prebiotic effects, mucus interaction and barrier support	In vitro fermentation, animal studies and human biomarkers	Structure–function relationships remain poorly characterised	[52,53]
Fermentation-derived peptides/GABA	Possible host signalling and indirect microbial effects	Metabolomics, receptor or clinical endpoints	Evidence is mainly mechanistic	[1,15]

**Table 5:** Health-Related Effects of Traditional Fermented Cereals.

Health Areas	Reported Outcome	Evidence Base	Overall Interpretation	Reference
Gastrointestinal health	Improved digestibility, pathogen inhibition, reduced diarrhoea risk, or improved bowel function	Mostly food microbiology, in vitro and observational evidence; limited controlled trials	Moderate mechanistic plausibility; clinical certainty remains low	[2,7]
Micronutrient bioavailability	Phytate reduction and improved iron, zinc, calcium or magnesium accessibility	Food chemistry, simulated digestion and some feeding studies	Bioaccessibility is more frequently measured than absorption or nutritional status	[2,54,55]
Glycemic and metabolic responses	Modified starch digestibility, organic-acid effects and SCFA-linked metabolic signalling	Acute meal tests, animal models and mechanistic studies	Product formulation and serving context strongly affect the metabolic response	[47,49,54]
Immune and barrier function	Potential modulation of cytokines, mucosal immunity and tight-junction integrity	Predominantly non-human or general probiotic evidence	Clinical evidence from Sub-Saharan African fermented cereals remains sparse	[7,15,46]
Cardiovascular outcomes	Potential ACE-inhibitory peptides, bile-salt hydrolase activity and lipid effects	In vitro assays and limited intervention evidence	Clinical cardiovascular endpoints are rarely measured	[15,56,57]
Antioxidant/anti-inflammatory effects	Release of phenolics and production of antioxidant metabolites	Chemical assays, cell models, and animal studies	Chemical antioxidant capacity should not be interpreted as direct evidence of human health benefit	[1,50]
Growth and complementary feeding	Improved digestibility, energy density, safety and acceptability	Infant-feeding and community nutrition studies	Nutrient dilution, inadequate fortification and microbial contamination risks should also be considered	[2,56]

**Table 6:** Research gaps and future priorities.

Priority	Recommended Action	Expected Contribution
Human intervention evidence	Randomised or well-controlled feeding studies using culturally realistic products and doses	Direct evidence of causality and clinical relevance
Strain-level validation	Whole-genome sequencing, safety assessment, dose, and shelf-life viability	Prevents overstatement of probiotic status
Microbiome functionality	Shotgun metagenomics, meta transcriptomics and fecal metabolomics	Moves beyond taxonomic description to biological function
Food-matrix standardisation	Report cereal variety, fermentation time, temperature, pH, starter/back-slopping and viable counts	Improves reproducibility and comparability
Precision-response analysis	Pre-specify modifiers such as baseline microbiome, age, diet, sex and metabolic phenotype	Identifies responders and supports stratified recommendations
Geographic coverage	Expand research beyond a small number of countries and document under-studied products	Improves regional representativeness
Safety and implementation	Assess pathogens, mycotoxins, antimicrobial resistance, acceptability and cost	Supports responsible translation and scale-up

## Data Availability

Data sharing is not applicable. No new data were created or analyzed in this study.

## Supplementary Materials

The following supporting information can be downloaded at: https://www.mdpi.com/article/10.3390/biology15181582/s1, PRISMA—ScR checklist form for Scoping Review.
