# Supplementary material for: Scoping Review on Traditional Fermented Cereals as Catalysts of Precision Nutrition: Mapping Evidence on Probiotic Activity, Gut Microbiota Modulation, and Human Health in Sub-Saharan Africa
Source: Biology (Basel). 2026 Sep 9;15(18):1582. doi: 10.3390/biology15181582 (PMC13603940; doi:10.3390/biology15181582)
Supplement: Supplementary file 1 [file biology-15-01582-s001.zip › biology-4479852-supplementary.pdf]

Preferred Reporting Items for Systematic reviews and Meta-Analyses extension for Scoping Reviews (PRISMA-ScR) Checklist

SECTION	ITEM	PRISMA-ScR CHECKLIST ITEM	REPORTED ON PAGE #
**TITLE**			
Title	1	Scoping Review on Traditional Fermented Cereals as Catalysts of Precision Nutrition: Mapping Evidence on Probiotic Activity, Gut Microbiota Modulation, and Human Health in Sub-Saharan Africa	1
**ABSTRACT**			
Structured summary	2	**Background:**<br>Traditional fermented cereals are widely consumed in Sub-Saharan Africa (SSA). These cereals provide beneficial microorganisms, fermentable substrates, and bioactive metabolites. However, evidence linking these foods with gut microbiota modulation, human health, and precision nutrition remains fragmented and varies in strength.<br>**Objectives:**<br>This scoping review aimed to map evidence linking traditional fermented cereals with probiotic functionality, gut microbiota modulation, and human health. It also examined their potential role in microbiome-informed precision nutrition in SSA. The review further identified geographical, methodological, and translational research gaps.<br>**Eligibility criteria:**<br>Human, animal, and in vitro studies were eligible. Studies had to examine traditional fermented cereal products from SSA or SSA-origin cereal products studied elsewhere. Eligible studies reported microbial, probiotic, gut microbiota, metabolite, or health-related outcomes. Only peer-reviewed original studies published in English were included. Editorials, conference abstracts, dissertations, and studies focused exclusively on non-cereal foods were excluded.<br>**Sources of evidence:**<br>Scopus, PubMed, Web of Science, ScienceDirect, and Google Scholar were searched from 2000 to 2026. Reference lists and forward citations of relevant studies and reviews were also examined.<br>**Charting methods:**<br>Data were independently charted or verified by two reviewers. The extraction form captured product, cereal substrate, fermentation method, study design, population, microbial identification methods, strains, probiotic assays, metabolites, gut microbiota outcomes, health outcomes, principal findings, and limitations etc,<br>**Results:**<br>The searches identified 1,284 database records and 56 additional records from other sources. After removing 318 duplicates, 1,022 records were screened. A total of 142 full-text reports were assessed, and 53 studies were included. Lactic acid bacteria and yeasts were the predominant microorganisms reported. Sorghum and millet were among the most widely studied cereals. Evidence was stronger for food-level microbial characteristics, probiotic-related properties, and improved nutrient bioaccessibility than for direct modulation of the gut microbiota and clinical health outcomes. Strain-level validation, dose definition, microbiome studies, and controlled human trials were uncommon.<br>**Conclusions:**<br>Traditional fermented cereals show promising nutritional, microbial, and functional properties. However, evidence remains insufficient to establish consistent effects on gut microbiota or human health. Their application in precision nutrition remains an emerging research area. Further well-designed human intervention studies, strain-level characterisation, and high-resolution microbiome and multi-omics investigations are needed.	1-5, 5, 6, 7, 8, 8-9, 10, 10-11, 2, 2

St. Michael's
Inspired Care.
Inspiring Science.

SECTION	ITEM	PRISMA-ScR CHECKLIST ITEM	REPORTED ON PAGE #
**INTRODUCTION**			
Rationale	3	**Rationale:** Existing reviews have examined fermented cereals, probiotics, and gut microbiota largely as separate topics, while evidence integrating these areas with human health and precision nutrition in Sub-Saharan Africa remains limited. This review, therefore, mapped the available evidence and identified key knowledge gaps. **Scoping review rationale:** A scoping review was appropriate because the evidence was broad, multidisciplinary, heterogeneous, and derived from diverse study designs. The objective was to map the breadth and nature of the evidence and identify research gaps, rather than to conduct a quantitative effectiveness synthesis.	5-6
Objectives	4	The review addressed traditional fermented cereal foods and cereal substrates (Population/Participants), probiotic functionality, microbial communities, gut microbiota, bioactive metabolites, and health outcomes (Concept), within Sub-Saharan Africa or involving SSA-origin cereal products studied elsewhere (Context). The objectives were to map the evidence on probiotic properties, gut microbiota modulation, health outcomes, and the potential integration of these foods into microbiome-informed precision nutrition.	5-6
**METHODS**			
Protocol and registration	5	Nil. The Scoping review does not require registration.	0
Eligibility criteria	6	Studies published from 2000 to 2026 were eligible. Peer-reviewed original studies published in English were included. Editorials, conference abstracts, dissertations, and studies focused exclusively on non-cereal foods were excluded. These criteria ensured that the evidence was relevant and accessible to the review objectives.	6
Information sources*	7	Information sources included PubMed, Scopus, Web of Science, ScienceDirect, and Google Scholar. Searches covered 2000–2026. Reference lists and forward citations of included studies and key reviews were also examined.	7
Search	8	The full electronic search strategy included Boolean search terms for traditional fermented cereals, probiotic microorganisms, gut microbiota, precision nutrition, and Sub-Saharan Africa.	6-7
Selection of sources of evidence†	9	Two reviewers independently screened titles and abstracts against the predefined eligibility criteria. Potentially relevant studies underwent full-text assessment by the same reviewers. Disagreements were resolved through discussion and consensus, and reasons for full-text exclusion were noted.	7
Data charting process‡	10	Data were charted, and two reviewers independently verified the data.	8
Data items	11	Data items included country, fermented cereal product, cereal substrate, fermentation method, study design, sample/population, microbial identification methods, microbial strains, probiotic assays, metabolites, gut microbiota outcomes, health outcomes, principal findings, and study limitations.	8
Critical appraisal of individual sources of evidence§	12	A formal critical appraisal was not conducted because the review aimed to map the breadth and nature of evidence rather than assess intervention effectiveness.	8-9
Synthesis of results	13	Charted data were summarised descriptively and thematically. Findings were organised by fermented cereal products, microorganisms, probiotic properties, gut microbiota effects, and health outcomes. Tables were used to present the mapped evidence.	10
**RESULTS**			
Selection of sources of evidence	14	A total of 1,284 database records and 56 additional records were identified. After removing 318 duplicates, 1,022 records were screened, and 872 were excluded. Of 150 reports sought for retrieval, 8 could not be retrieved. The remaining 142 full-text reports were assessed, with 89 excluded: wrong product (n=27), wrong outcome (n=25), wrong context (n=18), and other reasons (n=19). Finally, 53 studies were included.	11

SECTION	ITEM	PRISMA-ScR CHECKLIST ITEM	REPORTED ON PAGE #
Characteristics of sources of evidence	15	Study characteristics included cereal products and substrates, fermentation methods, study designs, populations, microorganisms and strains, probiotic assays, metabolites, gut microbiota and health outcomes, findings, and limitations.	8, 12–33
Critical appraisal within sources of evidence	16	No formal critical appraisal was conducted for the included studies.	8-9
Results of individual sources of evidence	17	For each included study, the charted data were presented according to the review objectives, including the fermented cereal product and cereal substrate, fermentation method, microorganisms and probiotic properties, metabolites, gut microbiota outcomes, health outcomes, and principal findings. These results were synthesised thematically across the studies included and presented in tables.	12-33
Synthesis of results	18	Charted data were summarised descriptively and thematically according to the review objectives. Findings were grouped by fermented cereal products, microorganisms, and probiotic properties, gut microbiota effects, metabolites, and health outcomes. Tables were used to map the evidence.	10
**DISCUSSION**			
Summary of evidence	19	The review identified evidence on traditional fermented cereals, probiotic microorganisms, gut microbiota modulation, metabolites, and health outcomes. Lactic acid bacteria and yeasts were the predominant microorganisms, while sorghum and millet were among the most studied cereals. Evidence was stronger for nutritional, microbial, and probiotic-related effects than for direct modulation of the gut microbiota and clinical health outcomes. The findings indicate promising potential for traditional fermented cereals in microbiome-informed precision nutrition, but further human and multi-omics studies are needed.	2, 12-33
Limitations	20	The review was limited by the English-language restriction, exclusion of grey literature, heterogeneity in study designs and outcome measures, and limited human evidence. Geographical representation across Sub-Saharan Africa was also uneven, with greater evidence from some countries than others. These limitations may have restricted the completeness and generalisability of the evidence base.	32-33
Conclusions	21	Traditional fermented cereals show promising nutritional, microbial, probiotic, and functional potential. However, evidence for consistent modulation of the gut microbiota, human health benefits, and precision nutrition applications remains limited. Further well-designed human studies, strain-level characterisation, and microbiome and multi-omics investigations are needed to strengthen the evidence and support future precision-nutrition applications in Sub-Saharan Africa	32
**FUNDING**			
Funding	22	Research and Publication Committee (RPC), Office of the Directorate of Research and Innovation, University of Venda, South Africa, for PDRF 48.	32

JBI = Joanna Briggs Institute; PRISMA-ScR = Preferred Reporting Items for Systematic reviews and Meta-Analyses extension for Scoping Reviews.

* Where *sources of evidence* (see second footnote) are compiled from, such as bibliographic databases, social media platforms, and Web sites.

† A more inclusive/heterogeneous term used to account for the different types of evidence or data sources (e.g., quantitative and/or qualitative research, expert opinion, and policy documents) that may be eligible in a scoping review as opposed to only studies. This is not to be confused with *information sources* (see first footnote).

‡ The frameworks by Arksey and O'Malley (6) and Levac and colleagues (7) and the JBI guidance (4, 5) refer to the process of data extraction in a scoping review as data charting.

§ The process of systematically examining research evidence to assess its validity, results, and relevance before using it to inform a decision. This term is used for items 12 and 19 instead of "risk of bias" (which is more applicable to systematic reviews of interventions) to include and acknowledge the various sources of evidence that may be used in a scoping review (e.g., quantitative and/or qualitative research, expert opinion, and policy document).

From: Tricco AC, Lillie E, Zarin W, O'Brien KK, Colquhoun H, Levac D, et al. PRISMA Extension for Scoping Reviews (PRISMAScR): Checklist and Explanation. Ann Intern Med. 2018;169:467–473. doi: 10.7326/M18-0850.
